# An exhaustive analysis of post-traumatic brain injury dementia using bibliometric methodologies

**DOI:** 10.3389/fneur.2023.1165059

**Published:** 2023-06-29

**Authors:** Xian-Zheng Sang, Cheng-Qing Wang, Wen Chen, Hong Rong, Li-Jun Hou

**Affiliations:** ^1^Department of Neurosurgery, The Second Affiliated Hospital of Naval Medical University, Shanghai, China; ^2^Department of Outpatient, The First Affiliated Hospital of Hainan Medical University, Haikou, China

**Keywords:** brain injuries, traumatic, dementia, chronic traumatic encephalopathy, Alzheimer disease, bibliometric

## Abstract

**Background:**

It is widely accepted that traumatic brain injury (TBI) increases the risk of developing long-term dementia, although some controversies surrounding this topic exist. Annually, approximately 69 million individuals suffer from TBI all around the world. Such a large population of TBI patients could lead to a future surge in the number of dementia patients. Due to the potentially severe consequences of TBI, various research projects on post-TBI dementia have emerged worldwide. Therefore, it is essential to comprehend the current status and development of post-TBI dementia for future research.

**Objective:**

The purpose of the study was to provide an overview of the field and identify hotspots, research frontiers, and future research trends for post-TBI dementia.

**Methods:**

Articles related to post-TBI dementia were retrieved from the Web of Science Core Collection for the period between 2007 and 2022, and analyzing them based on factors such as citations, authors, institutions, countries, journals, keywords, and references. Data analysis and visualization were conducted using VOSviewer, CiteSpace, and an online bibliometric platform (https://bibliometric.com).

**Results:**

From 2007 to 2022, we obtained a total of 727 articles from 3,780 authors and 1,126 institutions across 52 countries, published in 262 journals. These articles received a total of 29,353 citations, citing 25,713 references from 3,921 journals. Over the last 15 years, there has been a significant upward trend in both publications and citations. The most productive country was the United States, the most productive institution was Boston University, and the most productive author was McKee AC. *Journal of Neurotrauma* has been identified as the periodical with the greatest number of publications. Three clusters were identified through cluster analysis of keywords. A burst in the use of the term “outcome” in 2019 is indicative of a future research hotspot. The timeline view of references showed 14 clusters, of which the first 4 clusters collected the majority of papers. The first 4 clusters were “chronic traumatic encephalopathy,” “age of onset,” “tauopathy,” and “cognitive decline,” respectively, suggesting some areas of interest in the field.

**Conclusion:**

The subject of post-TBI dementia has raised much interest from scientists. Notably, America is at the forefront of research in this area. Further collaborative research between different countries is imperative. Two topical issues in this field are “The association between TBI and dementia-related alterations” and “chronic traumatic encephalopathy (CTE).” Studies on clinical manifestation, therapy, pathology, and pathogenic mechanisms are also popular in the field.

## Introduction

Traumatic brain injury (TBI), caused by striking, knocking, shaking the head, and so on, could lead to several harmful effects on various aspects of daily life, including executive capabilities, interpersonal relationships, mindset, behavioral modes, and learning abilities.^1^ Several researches suggested that TBI might be a risk factor for the development of dementia. Tagge et al. (1) found that closed TBI could induce acute and sustained impairment to axonal conduction velocity in the hippocampus. Stopa et al. (2) proposed that TBI patients had an increased risk of developing dementia. They conducted a retrospective cohort study involving 24,846 patients with a follow-up of 10 years and found that patients with TBI had a hazard ratio (HR) of 2.2 for developing dementia. Another cohort study conducted in Denmark by Osler et al. (3) supported Stopa et al.’s (2), highlighting an even stronger correlation between TBI and early-onset dementia (diagnosed at age 60 and before, HR 5.49). TBI patients may have a higher prevalence of Alzheimer’s disease (AD), Vascular dementia (VD), Parkinson’s disease (PD), and mild cognitive impairment (MCI) (4, 5). The 2020 report by the Lancet Commission expressly suggested that TBI could raise the possibility of the onset of dementia with a population attributable fraction (PAF) of 3.4% (6). In sum, it is widely accepted in mainstream literature that TBI is a significant risk factor for dementia despite some controversies in the field (7–10).

It is estimated that around 69 million people worldwide suffer from TBI annually (11). Such a large population of TBI patients might lead to a future surge in the number of dementia patients, with great implications for societies. As such, investigations into post-TBI dementia hold significant importance from both medical and societal standpoints. A thorough comprehension of the discipline’s current state and the progress of developments would be crucial for future studies. Additionally, identifying the pivotal literature and potential avenues of research could assist scholars in promptly ascertaining their research directions.

Bibliometrics is a discipline that assesses academic productivity using quantitative means, which provides plenty of statistical parameters, like publication count, citation count, H-index, and impact factor (IF) (12). The bibliometric analysis gives us an efficient means of grasping an overview of a given field, discerning its development trajectory, and highlighting notable scholars and work. Substantial aspects of the science domain could be described in the form of scientific networks, like the maps of co-authorship, co-occurrence, citation, and co-citation. These networks are beneficial to our intuitive understanding of the various changes that have taken place in reality (13). Bibliometric mapping is a momentous part of bibliometric analysis, and several tools available could assist us in mapping, like VOSviewer and CiteSpace. Bibliometric mapping could be divided into two parts: (1) the construction of maps and (2) the visualization of graphics (13). VOSviewer is a software designed for bibliometric network construction and visualization, with a particular focus on the graphical representation of maps. It is adept at the diagrammatic processing of large data sets efficiently (13). CiteSpace, developed by Chaomei Chen’s team, could visualize the network of co-citations and facilitate the exploration of the progression of subjects (14, 15). Bibliometric analyses, employing software tools, are widely utilized in the medical field (16–18).

Following our preliminary search, we have identified a rapid increase in the number of publications after 2007 in the discipline of post-TBI dementia (as shown in Supplementary Figure S1). Therefore, we made the bibliometric analysis based on publications about post-TBI dementia from 2007 to 2022 to gain insight into the current status and progress of this discipline. We intend to answer: What about the distribution of scientific productivity in this area? Which are the research hotspots in the field? What will be the future trends in the discipline? Our study aims to provide a quick panoramic view of the field to assist researchers in identifying areas of interest for further exploration.

## Method

### Data source

The Clarivate Analytic’s Web of Science Core Collection (WOSCC) database was selected as our primary data source. To ensure the accuracy and quality of retrieval, the Citation Index was set to Science Citation Index Expanded (SCIE), one of the subdatabases of WOSCC which is a comprehensive, multi-disciplinary database encompassing more than 8,600 specialized journals (17). Clarivate Analytic’s Web of Science (WOS), as one of the most influential platforms in terms of retrieval, access, and analysis of citations, has offered great support to scientific researchers in various fields and made tremendous contributions to the development of natural science. Moreover, there is a widespread application of WOS in the field of medicine (16).

### Search strategy

The retrieval formula was “TS = [(“traumatic brain injury” OR “chronic traumatic encephalopathy”) AND (“dementia” OR “Alzheimer’s disease”)].” “Year of publication” was set from 2007 to 2022. Only original researches were selected. The retrieval was made on October 12, 2022, and 1,590 documents were gained. The result was checked by Citespace to remove the duplication. The rest was scrutinized by three researchers to exclude articles that were unrelated to the topic or of which the primary content was not about post-TBI dementia. Lastly, a total of 727 articles were retained as the basis for the follow-on analysis. Supplementary Figure S2 depicts the flowchart of data acquisition.

### Data analysis

The related analysis was conducted based on Microsoft Excel 2019, CiteSpace 6.1.R2 (64-bit), VOSviewer 1.6.18, and an online analysis platform.^2^

Based on CiteSpace, we performed network analyses of the co-authorship of countries and institutions, and the co-citation of references. The parameters were set as follows: Time slice (2007 to 2022), Years per slice (1), Link (Strength: Cosine, Scope: Within Slices), and Selection Criteria (g-index: *k* = 25). VOSviewer was used to conduct network analyses of the co-authorship of organizations and authors, the co-occurrence of all keywords, the citation of sources, and the co-citation of cited authors. Each node in the map, generated by the above two pieces of software, represents a country, institute, author, or other subject, of which the size reflects the frequency of appearance. The edges represent the connection between nodes, and their thickness denotes the tightness of the association. And the colors of the nodes represent the clusters they belong to. Moreover, some indicators, such as total link strength (TLS) for VOSviewer and betweenness centrality (BC) for CiteSpace, measure the significance of a node within a given network (17, 18). Besides, when we implemented the co-citation analysis, the newly published papers were likely to be underestimated because of their relatively poor citations. Burst detection, which could recognize the emergent words no matter how many citations they had, is conducive to solving the problem (15). We conducted the keyword-burst detection analysis to catch some significant and emergent subject matters. In addition, we performed analyses of the distribution of annual publications across countries and international cooperation with the help of the web https://bibliometric.com. Microsoft Excel 2019 was used for data reorganization.

## Results

### General data

**Figure 1:**
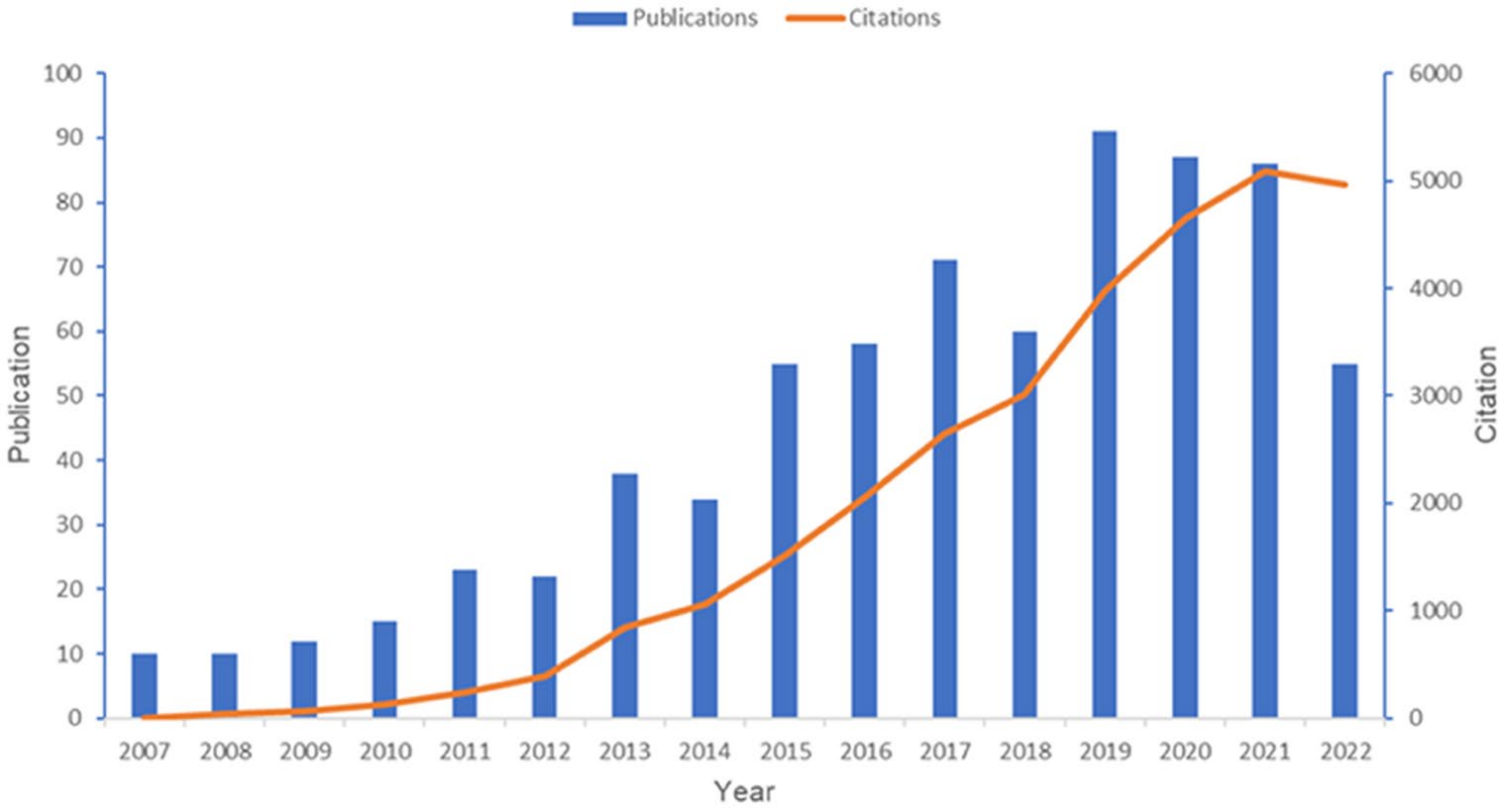
Overview of publications in the field of post-TBI dementia from 2007 to 2022. This figure is generated using Microsoft Excel 2019.

A total of 727 articles were retrieved, between 2007 and 2022, from 1,126 institutions and 52 countries, authored by 3,780 individuals, and published in 262 periodicals, which cited 25,713 references from 3,921 journals. Cumulatively, these publications have been cited 29,353 times. Figure 1 shows the publication and citation of papers in this field year after year. In general, the number of annual publications showed an upward trend despite the fluctuations from 2007 to 2019 and reached a stable level for the next few years (around 90 articles per year). Concerning the situation of citations, we could find a strong uptrend during the past 15 years. The above findings demonstrate the increased scholarly interest in post-TBI dementia in recent years, highlighting its position as a hotspot in the field of TBI.

### Countries and institutions analysis

**Figure 2:**
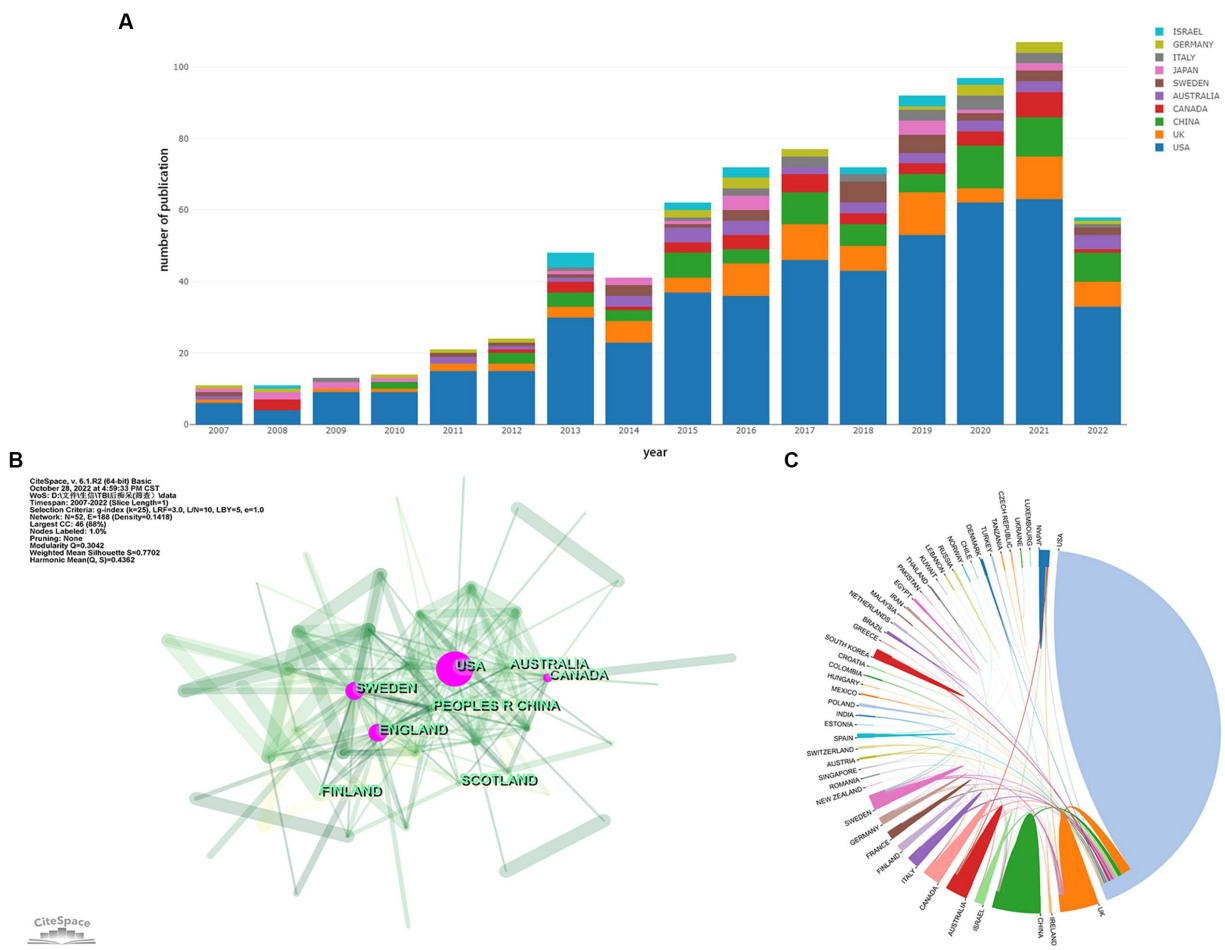
**(A)** Contribution of each country to the annual volume of publication. **(B)** Network map of countries/regions citation. **(C)** Visualization of international cooperation among countries/regions. The strength of cooperation is reflected in the thickness of the connection line. **(A,C)** are generated using https://bibliometric.com, while **(B)** is generated using CiteSpace.

We analyzed the contribution of each country to the annual number of publications, the result is shown in Figure 2A. It is apparent that American researchers participate in more than half of the papers issued each year from 2007 to 2022. Although more and more countries have engaged in research activities in this field, the USA remains the most prominent country in terms of publication output. Table 1 shows the status of the five countries with the most publications. It could be found that the majority of papers are issued by the top 5 countries reflecting the phenomenon of the Matthew Effect that does exist in this domain (19). America holds 67.7% of the total items (492/727). Furthermore, we analyzed collaboration patterns among countries/regions based on CiteSpace (Figure 2B). The value of BC reflects the significance of nodes in networks, which could assist to detect the crucial nodes. In general, “BC > 0.1” are viewed as the criterion to determine the importance, and nodes meeting the criterion would be shown with purple rings (17, 18). The USA plays a critical role in the cooperation network among countries/regions, with a centrality of up to 0.69. Canada (BC of 0.23) and England (BC of 0.11) are also influential in this field. Notably, Sweden holds the second highest centrality among countries (BC of 0.26) despite contributing only 30 papers. To ascertain Sweden’s contribution to the development of the subject, we studied these 30 papers. We found that five articles had over 100 citations and 11 papers had more than or equal to 50 citations. In 2012, Bell et al. (20) found that APOE could regulate the activity of mural cells to control the integrity of the cerebrovascular system via the CypA-NFκB-MMP9 pathway, and CypA was a key target in the therapy of APOE-induced neurovascular impairment and neuronal dysfunction. By the time of our search, cited frequency of this document had reached 802 times. Winblad et al. (21) illustrated the economic burden, epidemiology, risk factors, pathogenic mechanisms, and prevention of Alzheimer’s disease (AD) and other dementias (ADOD) in *Defeating Alzheimer’s disease and other dementias: a priority for European science and society*. The researchers pointed out the issues existing in this field of ADOD in European society and put forward individual solutions to address each concern. This paper had been cited 882 times, implicating its significant contribution to the field. Another thing that needed to be focused on is the situation in China. China has observed a surge in post-TBI dementia research in recent years, with 77 publications, taking the second spot in number, but with a relatively low centrality (0.03). To examine our findings, we also visualized the collaboration among countries/regions with the help of a platform, “Bibliometric.com” (Figure 2C), which confirmed that the USA, Sweden, the UK, and Canada are actively engaging with other countries in this field.

**Table 1:** The top 5 countries in this field in terms of the number of publications.

Rank	Country	Documents	Citations	Average citations/publications	Centrality
1	USA	492	23,798	48.4	0.69
3	CHINA	77	1,863	24.2	0.03
2	ENGLAND	61	3,379	55.4	0.23
4	CANADA	39	1,857	47.6	0.11
5	AUSTRALIA	34	1,573	46.3	0.01

**Figure 3:**
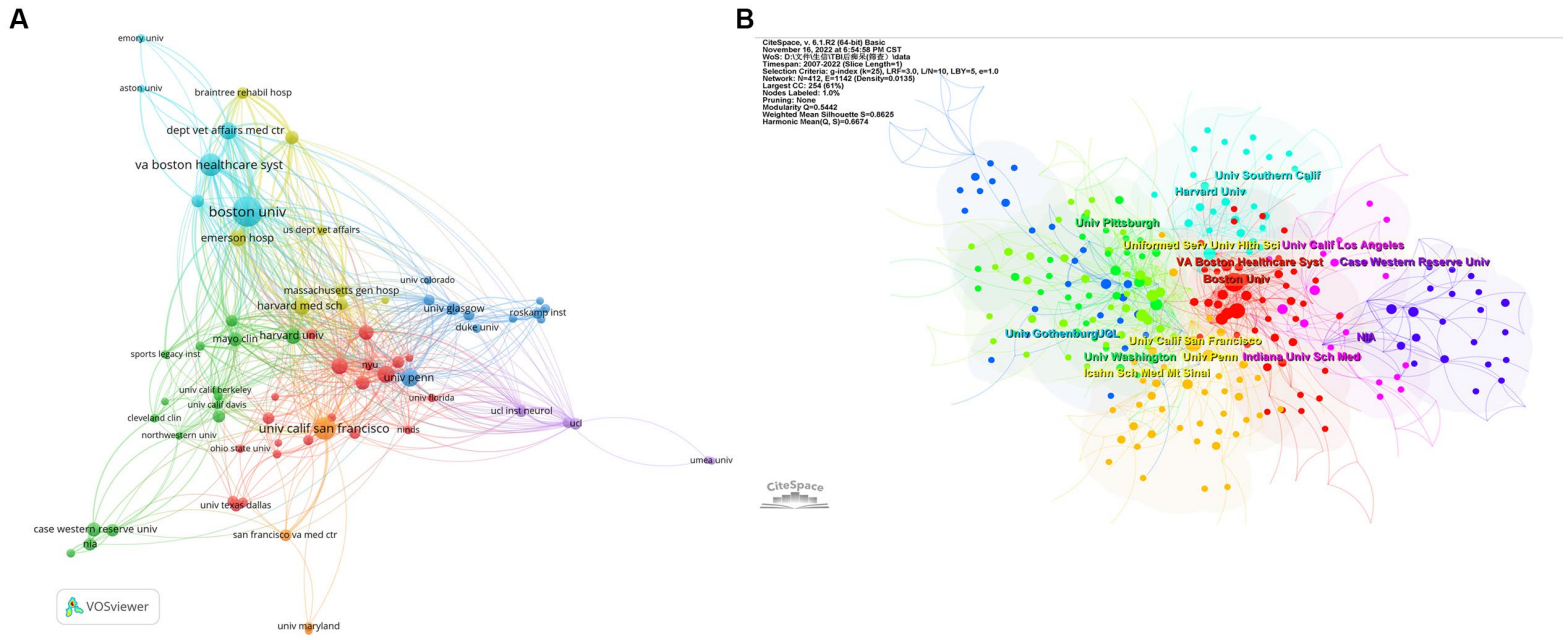
**(A)** Helicopter view of the co-authorship of the organizations. **(B)** Cluster view of co-authorship of organizations. **(A)** is generated using VOSviewer and **(B)** is generated using CiteSpace.

As for institutions, we analyzed the co-authorship of organizations by VOSviewer, as shown in Figure 3A. Boston University, which has published 85 articles, possesses the most notable academic influence in this field with a total link strength (TLS) of 383. Veterans Affairs Boston Healthcare System (TLS of 226), University of California San Francisco (TLS of 194), and Harvard Medical School (TLS of 192) also occupy crucial positions (Figure 3A). Table 2 shows the top 10 organizations with the largest number of articles. We also conducted a cluster analysis of institutions. The analysis identified nine clusters in total, of which seven are presented in Figure 3B. Some of the clusters had similar numbers of institutions. The red cluster, headed by Boston University and Harvard Medical School, consisted of 37 institutions, making it the largest cluster. And the orange cluster, headed by the University of California San Francisco and the Uniformed Services University of the Health Sciences, is the second-largest cluster with 29 institutes.

**Table 2:** The top 10 institutions in terms of the number of publications.

Rank	Label	Total link strength	Documents	Citations
1	Boston University	383	85	6,749
2	Veterans Affairs Boston Healthcare System	226	50	3,831
3	University of California San Francisco	194	50	2,430
4	University of Pennsylvania	150	31	2,395
5	Harvard Medical School	192	29	690
6	Department of Veterans Affairs Medical Center	158	29	1,417
7	Uniformed Services University of the Health Sciences	159	28	1,284
8	Emerson Hospital	169	26	3,221
9	University of Washington	179	25	1,614
10	Harvard University	142	23	2,728

### Bibliometric analysis of the journals

**Figure 4:**
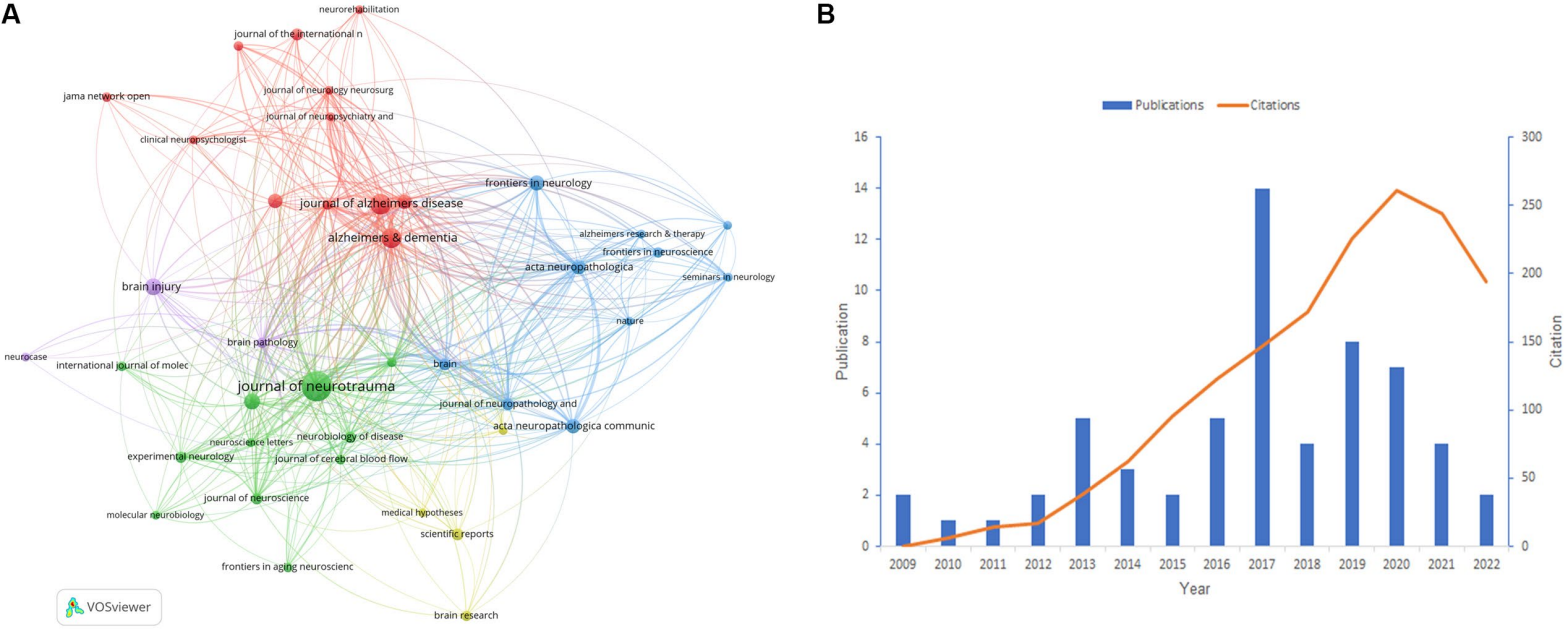
**(A)** Network analysis of the citation of journals. **(B)** The situation of publications and citations in the *Journal of Neurotrauma* from 2009 to 2022. **(A)** is generated using VOSviewer. **(B)** is created using Microsoft Excel 2019.

We conducted a statistical analysis of the periodicals in which the literature was published and found 262 journals in total. Table 3 shows the best 10 journals with the most papers in the field between 2007 and 2022. Notably, three journals had more than 25 articles: *Journal of Neurotrauma*, *Journal of Alzheimer’s Disease*, and *Alzheimer’s* & *Dementia*, with 60, 28, and 26 publications, respectively. Generally, the topics of the 10 journals involve Neuroscience, Clinical neurology, Rehabilitation, Pathology, and so on. Nine of the top 10 journals are specialized periodicals, except for *PLoS One*, which focuses on multidisciplinary science. *Alzheimer’s* & *Dementia* has the highest impact factor (IF) of 16.7, making it a leading journal in the field. Among the top 10 journals, eight are located in Q1 and Q2, demonstrating the high quality of research output. *Journal of Neurotrauma* is the only peer-reviewed journal concentrating on traumatic brain and spinal cord injury, and it covers a broad series of research types from basic biology to clinical trials [Journal of Neurotrauma: (liebertpub.com)]. Figure 4A depicts the network analysis of the sources of articles retrieved. Among the sources, the *Journal of Neurotrauma* attracts the most articles and also has the best TLS value of 283, demonstrating its great reputation in the field. We could find an irregular distribution of the annual publication volume of the *Journal of Neurotrauma* from 2009 to 2022, according to Figure 4B. However, the journal’s citation rate has shown consistent growth over the last decade or so.

**Table 3:** The top 10 journals with the largest number of documents.

Rank	Source	Documents	Citations	IF (2021)	JCR (2021)	Research area
1	Journal of Neurotrauma	60	1,530	4.9	Q2	NEUROSCIENCES AND CLINICAL NEUROLOGY
2	Journal of Alzheimer’s Disease	28	568	4.2	Q2	NEUROSCIENCES
3	Alzheimer’s and Dementia	26	1,482	16.7	Q1	CLINICAL NEUROLOGY
4	Brain Injury	19	245	2.2	Q4	NEUROSCIENCES AND REHABILITATION
5	Plos One	16	643	3.8	Q2	MULTIDISCIPLINARY SCIENCES
6	Frontiers in Neurology	15	127	4.1	Q2	NEUROSCIENCES AND CLINICAL NEUROLOGY
7	Journal of Head Trauma Rehabilitation	14	287	3.1	Q3	REHABILITATION AND CLINICAL NEUROLOGY
8	Neurology	14	759	12.3	Q1	CLINICAL NEUROLOGY
9	Acta Neuropathologica	13	1,058	15.9	Q1	NEUROSCIENCES AND CLINICAL NEUROLOGY AND PATHOLOGY
10	Acta Neuropathologica Communications	13	300	7.6	Q1	NEUROSCIENCES

### Bibliometric analysis of authors and co-cited authors

**Figure 5:**
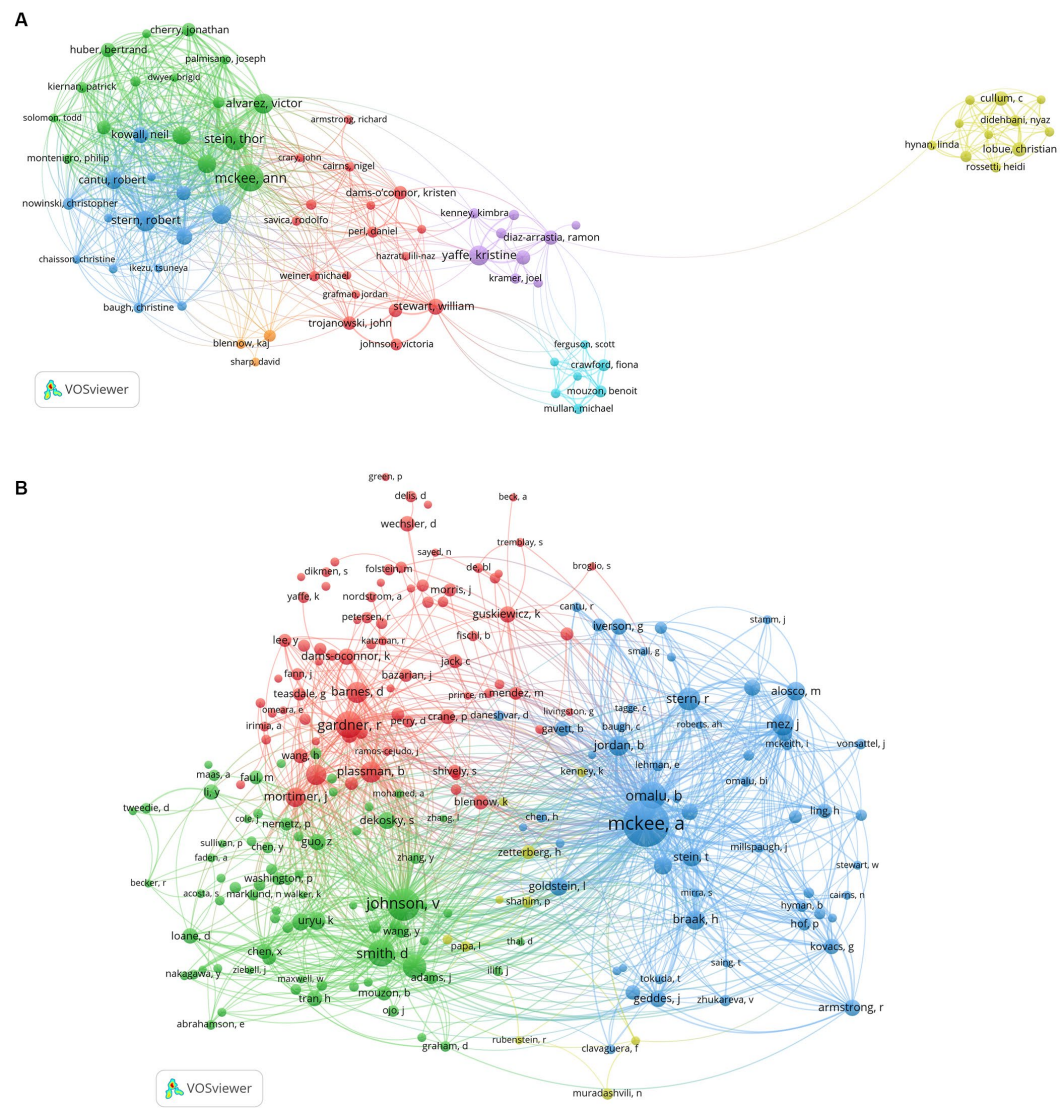
**(A)** The map of co-authorship of authors. **(B)** The map of co-citation of cited authors. **(A,B)** were performed on VOSviewer.

The study analyzed a total of 3,780 authors and 16,302 co-cited authors. We visualized the network analysis of the co-authorship of authors (Figure 5A) and the co-citation of cited authors (Figure 5B). Table 4 represents the 10 most productive authors and the top 10 co-cited authors with the best citations. The top 3 productive authors are McKee AC, Stein TD, and Stern RA, with 46, 33, and 28 papers, respectively. Interestingly, McKee AC holds first place both in terms of the number of publications and citations, who has 46 items, 5,418 citations, and an average citation rate of 117.8 citations per publication, making her the most prolific author in this discipline. Except for Yaffe, nine out of the top 10 productive scholars take office at Boston University, indicating the significance of this institution in the field. As for the co-cited authors’ network analysis, McKee AC, Johnson V, and Omalu B have the highest number of citations, and they also own the highest TLS scores, demonstrating their great contribution to this sector.

**Table 4:** The most productive authors in this field and the top 10 co-cited authors with the highest citations.

Rank	Author	Documents	Citations	Average citations/publication	Co-cited author	Citations	Total link strength
1	Mckee, AC	46	5,418	117.8	Mckee, AC	629	10,550
2	Stein, TD	33	3,377	102.3	Johnson, V	306	5,180
3	Stern, RA	28	3,691	131.8	Omalu, B	205	4,039
4	Yaffe, K	26	1,517	58.3	Gardner, R	204	3,063
5	Alvarez, VE	25	3,037	121.5	Smith, DH	204	3,833
6	Tripodis, Y	24	1,578	65.8	Roberts, G	159	3,045
7	Alosco, ML	21	752	35.8	Stern, RA	147	2,786
8	Mez, J	21	746	35.5	Mez, J	144	2,716
9	Cantu, R	20	2,889	144.5	Plassman, B	130	2,297
10	Kowall, N	16	2,458	153.6	Barnes, DE	128	1,765

### Network analysis of keywords

**Figure 6:**
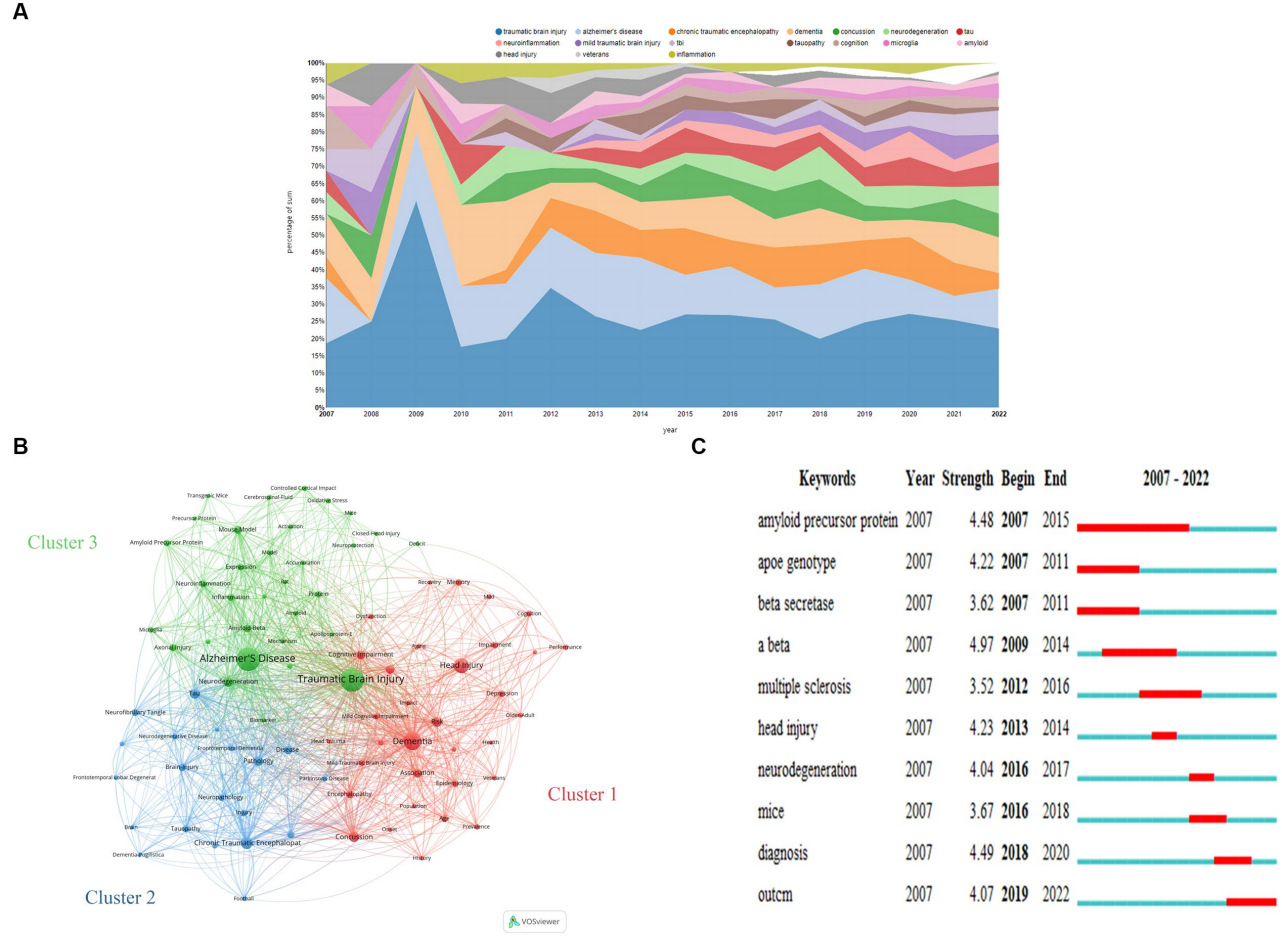
**(A)** Transition of keywords over the 15 years analyzed. **(B)** Network Visualization of keywords with a frequency of more than 16 in this field from 2007 to 2020 based on VOSviewer. **(C)** The top 10 keywords with the strongest citation bursts based on CiteSpace. **(A)** was obtained from https://bibliometric.com.

Figure 6A illustrates the evolution of keywords from 2007 to 2022. Notably, TBI and related terminology, Alzheimer’s disease, and dementia were prominent almost throughout the analyzed period. Additionally, chronic traumatic encephalopathy (CTE), concussion, neurodegeneration, tau, and neuroinflammation gained increased attention during the last decade. The top 5 keywords with the highest frequency are traumatic brain injury, dementia, Alzheimer’s disease, neurodegeneration, and tau-related terminology (*tau* and *tauopathy*) in 2022.

We generated a visual network of 85 frequently occurring keywords (frequency >16) across the 727 documents analyzed based on VOSviewer (Figure 6B). Through this, three distinct keyword clusters covering the years 2007 to 2022 were evident, and their specifics are shown as follows:

Cluster 1 encompasses a series of nouns outlining risk factors (e.g., *Concussion, Risk, Association, Risk-Factor, Encephalopathy, Age, Depression, Epidemiology, Mild Traumatic Brain Injury, Dysfunction, Prevalence, Older-Adult, Veterans, Impact, History, Population, Head Trauma, and Aging*), manifestation (e.g., *Cognitive Impairment, Encephalopathy, Memory, Depression, Impairment, Mild Cognitive Impairment, Cognition, Posttraumatic-Stress-Disorder, Dysfunction, Performance, and Cognitive Decline*), and therapy (e.g., *Rehabilitation, Recovery*).

Cluster 2 revolves around pathology and pathogenic mechanisms, covering a wide range of terms, such as *Alzheimer’s Disease, Neurodegeneration, Mouse Model, Amyloid-Beta, Neuroinflammation, Inflammation, Expression, Axonal Injury, Protein, Amyloid Precursor Protein, Microglia, Beta, Oxidative Stress, Diffuse Axonal, Injury, Activation, Mechanism, Biomarker, Accumulation, Neuroprotection, Cerebrospinal-Fluid, Deposition, Apolipoprotein-E, Precursor Protein, Amyloid, and Deficit.* It also encompasses terminologies about research models, such as *Controlled Cortical Impact, Model, Transgenic Mice, Mice, Rat, and Closed-Head Injury*.

Cluster 3 is centered on CTE, including terminologies such as *Chronic Traumatic Encephalopathy*, *Dementia-Pugilistica*, and terms related to CTE and other neurodegenerative diseases, such as *Tau*, *Pathology*, *Disease*, *Brain-Injury*, *Diagnosis*, *Tauopathy*, *Neurofibrillary Tangle*, *Neuropathology*, *Injury*, *Frontotemporal Dementia*, *Neurodegenerative Disease*, *Parkinson’s Disease*, *Amyotrophic-Lateral-Sclerosis*, *Frontotemporal Lobar Degeneration*, and *Degeneration*, as well as related issues, like *Football, National Institute*, and *Brain*.

Burst words are defined as keywords that emerge frequently within a given period, which imply research hot spots and development trends over time. Figure 6C shows the top 10 keywords with the strongest citation bursts.

The burst strength of the top 3 keywords, namely Aβ (amyloid β-protein), diagnosis, and amyloid precursor protein (APP), was found to be the highest. Specifically, Aβ, APP, and beta-secretase exhibited a citation burst between 2007 and 2015. Recently, scholars have placed greater value on diagnosis and outcome, making them the emerging focal points of the field. Other words that exhibited burst included apoe genotype (2007–2011), head injury (2013–2014), neurodegeneration (2016–2017), multiple sclerosis (2012–2016), and mice (2016–2018).

### Bibliometric analysis of references

**Figure 7:**
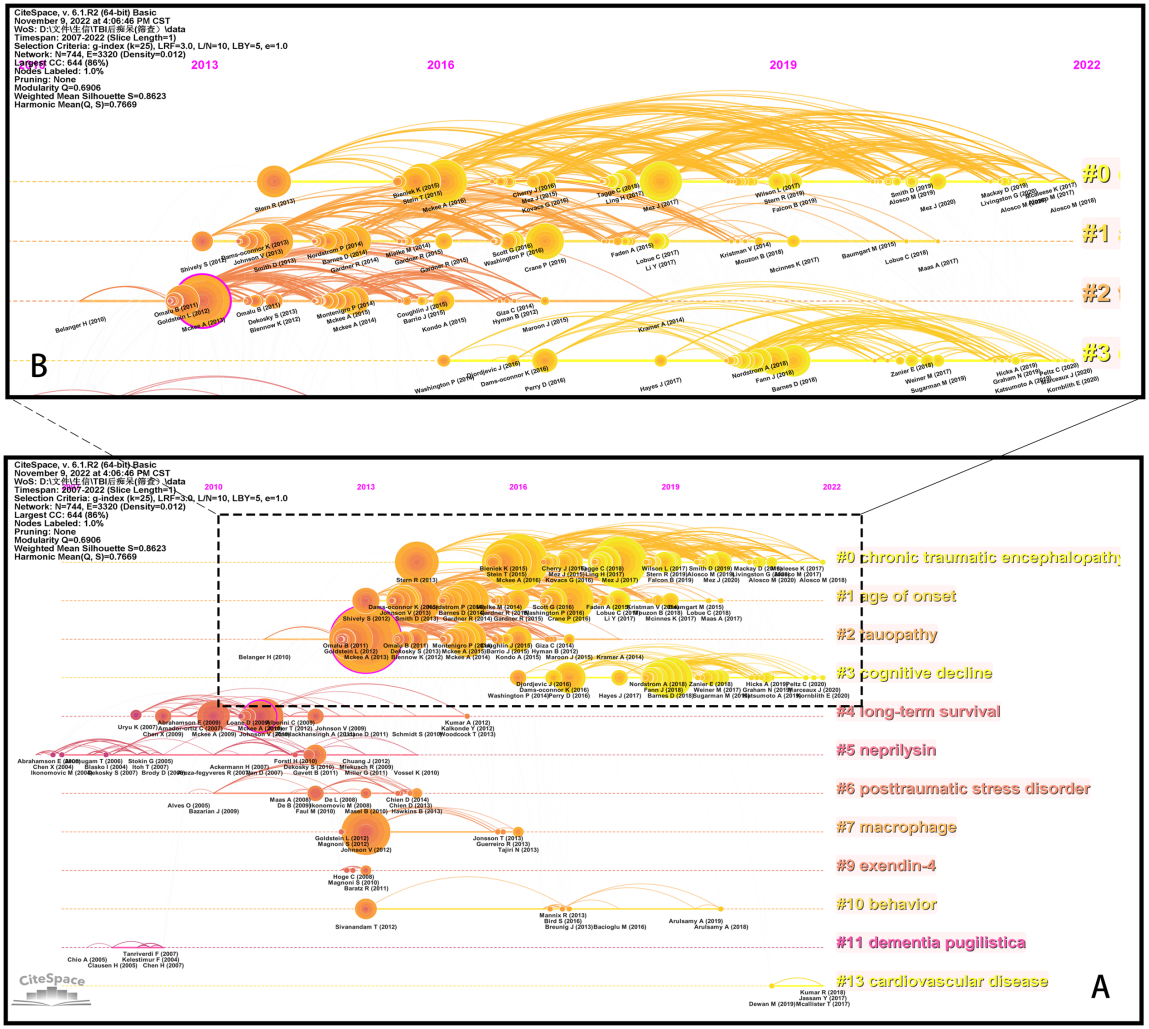
The timeline view of references. **(A)** Overview of 14 clusters. **(B)** Specifics of Clusters #0-#3 (dashed frame in **A**). The figure is obtained from CiteSpace.

Figure 7A depicts the timeline view of references cited in these articles, and reveals the presence of 14 clusters. The earliest cluster, identified as Cluster #5 *neprilysin*, emerged in 2007 and vanished in 2014. Cluster #4 *long-term survival* and Cluster #6 *posttraumatic stress disorder* represent the second and third earliest clusters, respectively. The largest one, Cluster #0 *chronic traumatic encephalopathy*, comprises 120 papers. And Cluster #13 c*ardiovascular disease* is the most recent emergence, with its first appearance in 2021. References from Clusters #0, #3, and #13 are still active to this day.

The paper titled “*TDP-43 proteinopathy and motor neuron disease in chronic traumatic encephalopathy*” (22) belongs to Cluster #4 and is notable with a high centrality of 0.13. It supplies the first evidence that repetitive traumatic brain injury (rTBIs) in contact sports might be associated with widespread TDP-43 proteinopathy and the onset and progression of motor neuron disease. Another bold piece of literature, belonging to Cluster #2, is “*The spectrum of disease in chronic traumatic encephalopathy*” (23), which has a centrality of 0.13 and the largest citation number of 103. In this paper, the researchers proposed a pathological staging of CTE and corresponding clinical manifestations based on evidence from postmortem brains and information from interviews with next of kin, which had greatly deepened our understanding of this disease.

We observed that the distribution of literature was imbalanced, with more than half of the references originating from the first four clusters (Figure 7A). Upon examining the citation trends over the years (as shown in Supplementary Figure S3), we made an interesting discovery. From 2007 to 2012, most of the cited references were concentrated in Clusters #4, #5, and #6. On the other hand, between 2013 and 2017, studies belonging to various clusters were cited. After 2018, the majority of citations were derived from the first four clusters (Clusters #0, #1, #2, and #3). Therefore, we focused our attention on Clusters #0, #1, #2, and #3 (Figure 7B) to track the kernel of the discipline at the moment. The labels of the 4 clusters are *chronic traumatic encephalopathy*, *age of onset*, *tauopathy*, and *cognitive decline*, respectively. We checked the high-frequency words in each cluster to investigate potential research frontiers (Table 5). Our findings reveal that common words, for example, *CTE*, *TBI-related*, *AD*, *tau-related*, *football*, and *cognition-related*, appear in most clusters if not all. These words mirror the shared research directions across the four classifications.

**Table 5:** Sketch of the first 4 clusters of references.

Cluster	keywords	Size	Span
#0	Chronic traumatic encephalopathy; traumatic brain injury; executive function; diagnostic validity; rater reliability | Alzheimer’s disease; amyloid beta peptides; microtubule-associated proteins; tau proteins; numerical data; chronic traumatic encephalopathy (22.53); football (13.29); proteome (10.33); extracellular vesicles (10.33); neurodegenerative disease (9.84)	120	Date from 2014
#1	Traumatic brain injury; Alzheimer’s disease; cognitive decline; normal cognition; risk factor | chronic traumatic encephalopathy; neurodegenerative disorders; apoe4; cortical thickness; neurodegeneration; age of onset (12.27); tauopathy (9.78); chronic traumatic encephalopathy (9.18); football (7.3); controlled cortical impact (6.12)	105	2013 ~ 2020
#2	Chronic traumatic encephalopathy; neurofibrillary tangles; principal components analysis; astrocytic tangles; monoacylglycerol lipase | traumatic brain injury; Alzheimer’s disease; risk factors; head injury; memory loss; chronic traumatic encephalopathy (15.71); tauopathy (9.24); concussion (8.71); neurodegenerative disorders (6.41); brain trauma (4.9)	102	2011 ~ 2018
#3	Traumatic brain injury; chronic traumatic encephalopathy; repetitive head impacts; animal models; mild behavioral impairment | Alzheimer’s disease; Parkinson’s disease; amyotrophic lateral sclerosis; soccer ball; seizure disorder; chronic traumatic encephalopathy (13.28); cognitive decline (8.92); traumatic brain injury (5.77); football (5.07); axonal injury (4.39)	82	Date from 2016

Additionally, exclusive terminologies were identified for each cluster. Cluster #0 exhibits unique content such as *Diagnostic validity*, *rater reliability*, *amyloid beta peptides*, *numerical data*, *proteome*, and *extracellular vesicles*. The terms specific to Cluster #1 include *apoe4*, *cortical thickness*, *age of onset*, and *controlled cortical impact*. Cluster #2 focuses on exclusive words such as *neurofibrillary tangles*, *principal components analysis*, *astrocytic tangles*, and *monoacylglycerol lipase*, while Cluster #3 incorporates terminologies such as *repetitive head impacts*, *animal models*, *Parkinson’s disease*, *amyotrophic lateral sclerosis*, *seizure disorder*, and *axonal injury*.

We also explored the top articles in the four clusters. Table 6 shows the top 5 papers with the most citations in four clusters. The specifics of the top 5 papers in each cluster are shown in Table 7.

**Table 6:** The top 5 papers from the first 4 clusters, respectively.

Cluster	Rank	Title	Count	References	Journal	Year of publication	Year of the first citation	Year of the last citation	Centrality
0	1	The first NINDS/NIBIB consensus meeting to define neuropathological criteria for the diagnosis of chronic traumatic encephalopathy	82	Mckee et al. (24)	ACTA NEUROPATHOL	2016	2016	2021	0.09
	2	Clinicopathological Evaluation of Chronic Traumatic Encephalopathy in Players of American Football	67	Mez et al. (25)	JAMA-JAM MED ASSOC	2017	2018	2022	0.07
	3	Beta-amyloid deposition in chronic traumatic encephalopathy	47	Stein et al. (26)	ACTA NEUROPATHOL	2015	2016	2020	0.04
	4	Clinical presentation of chronic traumatic encephalopathy	38	Stern et al. (27)	NEUROLOGY	2013	2014	2018	0.05
	5	Chronic traumatic encephalopathy pathology in a neurodegenerative disorders brain bank	37	Bieniek et al. (28)	ACTA NEUROPATHOL	2015	2016	2020	0.03
1	1	Association of Traumatic Brain Injury With Late-Life Neurodegenerative Conditions and Neuropathologic Findings	43	Crane et al. (29)	JAMA NEUROL	2016	2017	2021	0.08
	2	Dementia risk after traumatic brain injury vs. nonbrain trauma: the role of age and severity	27	Gardner et al. (30)	JAMA NEUROL	2014	2015	2019	0.04
	3	Chronic neuropathologies of single and repetitive TBI: substrates of dementia?	29	Smith et al. (31)	NAT REV NEUROL	2013	2014	2018	0.05
	4	Traumatic brain injury and risk of dementia in older veterans	38	Barnes et al. (32)	NEUROLOGY	2014	2015	2019	0.07
	5	Traumatic brain injury and young onset dementia: a nationwide cohort study	24	Nordstrom et al. (33)	ANN NEUROL	2014	2015	2019	0.03
2	1	The spectrum of disease in chronic traumatic encephalopathy	103	Mckee et al. (23)	BRAIN	2013	2013	2018	0.13
	2	Chronic traumatic encephalopathy in blast-exposed military veterans and a blast neurotrauma mouse model	42	Goldstein et al. (34)	SCI TRANSL MED	2012	2013	2017	0.06
	3	The neuropathology of sport	35	Mckee et al. (35)	ACTA NEUROPATHOL	2014	2015	2019	0.04
	4	The neuropathology of chronic traumatic encephalopathy	30	Mckee et al. (36)	BRAIN PATHOL	2015	2015	2020	0.01
	5	Clinical subtypes of chronic traumatic encephalopathy: literature review and proposed research diagnostic criteria for traumatic encephalopathy syndrome	28	Montenigro et al. (37)	ALZHEIMERS RES THER	2014	2015	2019	0.04
3	1	Association of Mild Traumatic Brain Injury With and Without Loss of Consciousness With Dementia in US Military Veterans	49	Barnes et al. (38)	JAMA NEUROL	2018	2019	2022	0.03
	2	Long-term risk of dementia among people with traumatic brain injury in Denmark: a population-based observational cohort study	36	Fann et al. (39)	LANCET PSYCHIAT	2018	2019	2022	0.03
	3	Association of traumatic brain injury with subsequent neurological and psychiatric disease: a meta-analysis	29	Perry et al. (5)	J NEUROSURG	2016	2017	2021	0.06
	4	Traumatic brain injury and the risk of dementia diagnosis: A nationwide cohort study	26	Nordstrom et al. (40)	PLOS MED	2018	2019	2022	0.01
	5	Traumatic Brain Injury and Alzheimer’s Disease: The Cerebrovascular Link	23	Ramos-Cejudo et al. (41)	EBIOMEDICINE	2018	2019	2022	0.01

**Table 7:** The profile of the top 5 papers from the first 4 clusters.

Cluster	Rank	Title	Profile
0	1	The first NINDS/NIBIB consensus meeting to define neuropathological criteria for the diagnosis of chronic traumatic encephalopathy	Defined the neuropathological criteria for the diagnosis of CTE
	2	Clinicopathological Evaluation of Chronic Traumatic Encephalopathy in Players of American Football	Postmortem of brain of former American football players suggesting the relationship between CTE and history of football-playing
	3	Beta-amyloid deposition in chronic traumatic encephalopathy	The deposition of Aβ was altered and accelerated in patient of CTE compared with normal aging people Aβ was related to severity of CTE independent of age
	4	Clinical presentation of chronic traumatic encephalopathy	Introduced two major kinds of manifestation of CTE, that is “behavior/mood variant” and “cognitive variant”
	5	Chronic traumatic encephalopathy pathology in a neurodegenerative disorders brain bank	Reported a few cases with concomitant CTE pathology and neurodegenerative diseases Found CTE pathology was only detected in those people with the history of contact sports
1	1	Association of Traumatic Brain Injury With Late-Life Neurodegenerative Conditions and Neuropathologic Findings	Studied the relationship between TBI with loss of consciousness (LOS) and neurodegenerative diseases as well as neuropathological alternation Found TBI with LOS had something to do with increased risk for Lewy body accumulation, PD, progression of parkinsonian signs and microinfarcts, but might have nothing to do with dementia, AD, neurofibrillary tangles and neuritic plaques
	2	Dementia risk after traumatic brain injury vs. nonbrain trauma: the role of age and severity	Investigated the link between TBI and dementia Demonstrated that younger adults were more tolerated with mild TBI than older people in term of onset of dementia
	3	Chronic neuropathologies of single and repetitive TBI: substrates of dementia?	A review about chronic neuropathological changes post TBI
	4	Traumatic brain injury and risk of dementia in older veterans	Identified positive relationship between TBI and development of long-term dementia in older veterans
	5	Traumatic brain injury and young onset dementia: a nationwide cohort study	Proposed that TBI of different severity might increase risk of young onset dementia (YOD) of non-AD forms
2	1	The spectrum of disease in chronic traumatic encephalopathy	Proposed the pathological staging of CTE and corresponding clinic manifestation based on evidences from postmortem brain and information from interviews with next of kin
	2	Chronic traumatic encephalopathy in blast-exposed military veterans and a blast neurotrauma mouse model	Discovered the existence of CTE pathology in postmortem brains from blast-exposed veterans Introduced a blast neurotrauma mouse model
	3	The neuropathology of sport	A review mentioning the sport-related adverse neuropathological changes including CTE
	4	The neuropathology of chronic traumatic encephalopathy	A review about CTE
	5	Clinical subtypes of chronic traumatic encephalopathy: literature review and proposed research diagnostic criteria for traumatic encephalopathy syndrome	Reviewed the clinical manifestation of CTE Suggested diagnostic criteria for research of CTE and related disorders
3	1	Association of Mild Traumatic Brain Injury With and Without Loss of Consciousness With Dementia in US Military Veterans	A cohort study investigating the association of different severity of TBI, especially mild TBI with or without LOS, with dementia
	2	Long-term risk of dementia among people with traumatic brain injury in Denmark: a population-based observational cohort study	A cohort study exploring the relationship between TBI and dementia Found TBI could increase the risk of dementia in a seemingly dose-dependent way, that is the risk of dementia raise with augmentation of the number of TBI
	3	Association of traumatic brain injury with subsequent neurological and psychiatric disease: a meta-analysis	Supported that there was a strong relationship between prior TBI and long-term neurological and psychiatric diseases Proclaimed that there was no evidence that more TBI could induce higher risk of disease
	4	Traumatic brain injury and the risk of dementia diagnosis: A nationwide cohort study	Supported the kind of “dose-dependent” relationship, found that more severe and multiple TBI could induced higher risk of dementia diagnosis Noticed risk decreased over time post TBI, although it was still notable even though more than 30 years after assault
	5	Traumatic Brain Injury and Alzheimer’s Disease: The Cerebrovascular Link	A review focusing on cerebrovascular connection between TBI and AD

## Discussion

Over the past 15 years, there has been a consistent increase in publications and citations pertaining to studies on post-TBI dementia. The bibliometric analysis allows for the visualization of several quantitative indicators and facilitates a more intuitive comprehension of the literature as compared to systematic reviews (17). In this study, we employed bibliometric analysis to examine the discipline of post-TBI dementia from various perspectives, including authors, journals, countries, institutions, keywords, and references, to gain a comprehensive understanding of research trends in this field globally and identify potential avenues for future development.

### General information

In this study, a total of 727 articles, between 2007 and 2022, from WOSCC were analyzed, which came from 1,126 institutions and 52 countries, authored by 3,780 authors, issued in 262 journals, and cited 25,713 references from 3,921 journals. Based on our study, it is evident that the discipline of post-TBI dementia has seen a steady development over the past 15 years, with increasing international participation. The United States, with a centrality of 0.69, is, without doubt, the most influential country in this field, contributing 67.7% of openly published papers. Moreover, all 10 top institutions are located in the USA, reinforcing America’s dominance as a leader in this domain (Table 2). Our findings also demonstrate that Chinese scholars have shown considerable interest in the subject of post-TBI dementia, with their number of research publications ranking second. Nevertheless, despite China’s conspicuous number of publications, its centrality ranking was relatively low indeed, indicating the need for strengthened academic cooperation and influence. It is imperative for China to conduct more high-quality research to reinforce its standing in this field. Our study also highlights the noteworthy research capabilities and robust collaboration of Sweden, which, despite having published only 30 papers, exhibits the second-highest centrality in the field.

Among the 10 top organizations, Boston University played a pivotal role in the study of post-TBI dementia, manifesting the highest number of articles and the highest TLS of 383. Other institutions, like the University of California San Francisco and Harvard University, also emerged as significant contributors to the development of the subject. In contrast to the country analysis, our network analysis showed no dominant institute, and focusing on different research directions, institutes form alliances with each other, leading to the formation of various clusters throughout the field. Almost all clusters of institutions were headed by American institutes, further suggesting that America dominates the field. Grasping the information about the authors is conducive to answering a significant question: Who are the pivotal authors? Our analysis demonstrates that the most well-known author in the area is McKee AC, with 5,418 citations, followed by Stern RA, Stein TD, Alvarez VE, Tripodis Y, and Yaffe K, all of whom are eminent scientists known for their remarkable productivity. McKee AC is no doubt the leading scientist in this domain, who has focused on AD and CTE constantly. She has an incredible interest in the pathology of neurodegenerative diseases, the action of tau, axon impairment, trauma, and vascular dysfunction. She has also conducted extensive researches on mild TBI (mTBI), induced by contact sports and military services, as well as its long-term effects [Ann C. McKee | Graduate Medical Sciences (bu.edu)]. She has contributed to over 70% of CTE cases reported to date [Ann McKee, MD | CTE Center (bu.edu)]. Of the top 10 productive authors, 5 are affiliated with Boston University, which demonstrates the contribution and influence of this institute. Analysis of journals could assist us to determine the distribution of literature across journals, identify the “core periodicals,” and provide some guidelines for manuscript submission. *Journal of Neurotrauma* ranks first in terms of the number of publications, followed by *Journal of Alzheimer’s Disease* and *Alzheimer’s & Dementia*.

### Hot spots and Frontiers

Keywords represent the core of a paper, assisting readers to understand quickly what the papers are about and which domain the articles pertain to. The transition of keywords could embody the development of subjects. Our analysis revealed that during the past decade, several terms, including CTE, concussion, neurodegeneration, tau, and neuroinflammation, had been frequently referenced in the literature. Additionally, TBI and TBI-related terminologies, as well as AD and dementia, have been identified as current hot spots in this field. Furthermore, co-occurrence analysis of keywords enables the identification of research frontiers. We observed three distinct clusters related to post-TBI dementia through a keywords network analysis, namely: (1) terms about risk factors, manifestation, and therapy; (2) terminologies relevant to pathology, pathogenic mechanisms, and models; and (3) CTE-related terminologies.

Mainstream viewpoints to date support TBI as a risk factor for dementia (4, 6, 10). Stopa et al. (2) conducted a retrospective cohort study with a follow-up period of greater than 10 years and found a positive relationship between TBI and dementia. Another cohort study held by Osler et al. (3) also supported this kind of association. Many meta-analyses had also drawn conclusions agreeing with the above studies (4, 5). However controversy still existed (7). It might be reasonable to say that not all TBIs are associated with an increased risk of developing dementia. One important consideration when planning research on post-TBI dementia is the variability of injury parameters [e.g. frequency (5)severity (2, 4)] and patient states [e.g. age (2, 4) and genotype (42)] across different settings. Concerning treatment, statins might become a potential medication for TBI patients. Redelmeier et al. (43) found that statins could decrease the risk of dementia in patients with a concussion. Atorvastatin seems to improve the prognosis of mild and moderate TBI patients (44). Li et al. (45) found that the combined use of angiotensin-converting enzyme inhibitors (ACEI) and statins could reduce the risk of possible AD in TBI patients. Further large-scale clinical trials should be initiated to authenticate the findings and their generalizability

Despite the growing interest in academia, the underlying mechanisms of post-TBI dementia remain unclear. The hypotheses in this regard are multi-factorial, comprised of deposition of pathological proteins, cognition reserve, chronic inflammation, chronic activation of microglia, degeneration of synaptic junctions induced by the alteration of protein degradation, excitotoxicity, overload of calcium ions, dysfunction of the mitochondrion, etc. (46, 47). Various TBI models have been employed in related studies, namely, shock wave tubes, Controlled Cortical Injury (CCI), Weight Drop Injury (WDI), and Fluid Percussion Injury (FPI), among others. CCI is used for focal injuries, while shock wave tubes are for diffuse injuries. WDI could cause either focal or diffuse injuries, depending on the experimental parameters chosen. Generally, mild WDI is relevant for diffuse injuries, while severe WDI could cause focal contusions. On the other hand, FPI could produce diffuse or mixed injury by setting different impulse pressures in experiments. FPI has been identified as the most dependable, repeatable, and practical model for blast injury (48). Furthermore, cognition impairment existing in open TBI is different from that in closed head injury even if the physiological condition is kept constant (48). Thus, the choice of appropriate models to mimic the injuries of reality is essential for research.

The contributions of many parties have led to CTE becoming a focal point in the field of neurotrauma. In 1928, Martland (49) introduced the disease of CTE to describe neuropsychiatric manifestations in pugilists – known as “punch drunk” at that time. This syndrome was later referred to as “dementia pugilistica” and is currently recognized as CTE (50). Nowadays, the application scope of the terminology is not limited to boxing but to extensive settings, such as football players (51). Omalu et al. (51) reported a case of CTE in a former National Football League player, arousing both academic and public concern about the neurological sequelae of contact sports. The film “*Concussion*,” adapted according to the experiences of Bennet Omalu, raised public awareness of this kind of neurodegenerative disease. The discovery of CTE has affected the real world deeply, raising emphasis on rTBIs in scientific, sports, and public contexts. The studies on CTE were also infested with controversy, as was the case with TBI (50), and further studies are necessary to fully understand the disease.

In co-citation and co-cited analyses, we may overlook the newly published papers that have not got enough attention. The detection of burst words is a way to solve the problem (15). Figure 6C illustrates that Aβ, APP, and β-secretase experienced a burst in popularity, between 2007 and 2015. Aβ, a kind of polypeptide of 38–43 amino acids, is formed by the decomposition of APP with the help of β-secretase and γ-secretase (47). Aβ has attracted strong interest from neuroscientists due to its visible manifestation in the pathology and pathogeny of AD. There seems to be a linkage between TBI and Aβ. Some scholars had found that TBI might increase the level of deposition of Aβ in the global, frontal cortex, and posterior cingulum (52). Further studies are needed to fully understand the role of Aβ in post-TBI dementia. In recent years, attention among researchers has gradually shifted to the prognosis and diagnosis of the disease. As for prognosis, numerous epidemiological studies are springing up, aiming to reveal cognition alteration and the onset of dementia following TBI (3, 8, 9). In respect of diagnosis, studies about biomarkers have become a trending topic. Except for Aβ, tau, which could regulate the elongation and stability of microtubes, has received great attention. The function of tau depends on its level of phosphorylation. And TBI could disturb the stability of microtubes and the activity of the nervous system by changing the level of phosphorylation of tau (53). Moreover, as a characteristic of the pathology of CTE, tau shows an immense practical perspective for diagnosis (24). Other potential diagnosed indicators include α-synuclein, TDP-43 (TAR DNA binding protein-43), the light chain of neurofilament, and cavum septum pellucidum (CSP) (54, 55). In addition, the keywords “multiple sclerosis” and “mice” also demonstrated a burst phenomenon (Figure 6C). The prominence of multiple sclerosis suggested that multiple sclerosis might be one of the potential complications post-TBI and received great interest from scholars worldwide. As was common in life science research, mice were frequently used as animal models in the research field of post-TBI dementia, having strongly contributed to the development of this discipline. Notably, the appearance and extinction of burst words may not always accurately mirror the development of a subject. For instance, the word “Apo E” (Apolipoprotein E) burst from 2007 to 2011 but was not detected after 2011, as shown in Figure 6C. Nevertheless, plenty of studies about Apo E sprout continuously, appealing to scientists in many fields. Apo E, which plays a pivotal role in the transportation of lipids in the central nervous system, has three types of alleles that correspond to encoding three kinds of proteins: E2, E3, and E4 (56). Especially, E4 is viewed as a risk factor for poor prognosis after TBI and contact sports (42), which might be a key target of post-TBI dementia. Therefore, the interpretation of the developmental trend of subjects is the result of an integrated analysis of an assortment of data.

References, cited by periodicals, constitute the research base of the subjects studied. The analysis of references (research bases) cited in research articles could reveal the evolution of research frontiers and the changes in disciplinary trends. The Cluster #5 Neprilysin was the earliest cluster. Neprilysin (NEP) is considered an ectoenzyme that could catalyze the proteolysis of several substrates, for example, enkephalins and tachykinins. Moreover, NEP might be a potential target of AD therapy, which was thought to be one of the primary amyloid-degrading enzymes. Many studies have revealed the role of NEP in cognitive function. Reduced levels of NEP expression and activity were identified in the cortex of elderly AD patients. Upregulation of NEP expression might alleviate the AD-like symptom (57). However, Maigler et al. (58) found that NEP or NEP2 deficiency seemed not to aggravate impairments in spatial learning or memory post-TBI. In some cases, NEP (NEP or NEP2) deficiency may have actually been protective. This finding contradicts previous research on the role of NEP in cognitive function and highlights the need for further investigation to reconcile this controversy.

Our analysis of the timeline view of cited references shows that the first 4 clusters were the primary sources of citations (Figure 7A). Furthermore, our examination of the evolution of references over time revealed a notable pattern (Supplementary Figure S3). From 2007 to 2012, the majority of references were clustered within three groups (Cluster #4, #5, and #6). However, from 2013 to 2017, research within this timeframe was widely dispersed and lacked a concentrated focus on any particular cluster. Beginning in 2018, a discernible trend toward established research directions in the area of post-TBI dementia emerged, characterized by Clusters #0, #1, #2, and #3. Overall, these findings reflect the natural process of scientific progress. As time went on, the research in this subject evolved and developed, moving from its early stages where knowledge was limited and only very limited domains can be explored, to a more advanced and complex understanding of the topic. Along the way, researches made “new” discoveries, which resulted in the exploration of different domains within the field. This led to a proliferation of studies across the entire spectrum of the discipline, as scientists chased the research frontiers. Eventually, with the growth of knowledge, a credible and promising mainline emerged, attracting the attention of the majority of scholars in the field.

Therefore, our focus shifted to the four clusters (Figure 7B). It seems that though these clusters share several common research themes, each also cultivates unique directions of inquiry. For instance, Cluster #0 appears to prioritize the diagnosis of the disease, while Cluster #1 centered on investigating the disease’s associated risk factors. In contrast, Cluster #2 examines pathology and pathophysiology within the context of the disease, and Cluster #3 might be earmarked for studies exploring the associations between TBI and various other diseases. To substantiate our hypothesis, we conducted a thorough examination of the influential articles in each cluster. We find a striking resemblance and considerate overlap in the first 4 clusters despite being separate clusters according to the CiteSpace algorithm. These clusters predominantly revolve around the correlation between TBI and dementia-related changes, along with knowledge concerning CTE, thereby indicating that epidemiological studies about causality are influential and highly sought after in this field, alongside studies concerning CTE. And the pathology of TBI and CTE is also a critical topic in this field. The aforementioned observations lend further support to the findings obtained from the cluster analysis of keywords.

It is worth noting that Cluster #13 *cardiovascular disease* appears to be a relatively new area of research, which may suggest a future research subfield. Eric Nyam et al. (59) found that TBI patients had increased risks of the onset of “major cardiovascular and cerebrovascular events” (MACCE), with the highest incidences within the first year following the diagnosis of TBI. Kumar et al. (60) found that hypertensive disease was one of the most prevalent comorbid conditions among TBI patients 55 years of age and older. Hammond et al. (61) discovered that hypertension and high blood cholesterol were prevalent comorbidities diagnosed synchronously with or after TBI, especially among people older than 50 years at the end of the follow-up, despite the possibility that the incidence of these two comorbidities seemed to be more relevant to aging than TBI. Combined with the fact that traumatic microvascular injury was considered one of the potential pathogenic mechanisms of chronic sequelae post-TBI (1), research on the cardiovascular and cerebrovascular alterations after TBI might become a future hotspot in this discipline.

When we searched papers on the bibliometrics of post-TBI dementia, we noticed an article, *Bibliometric Analysis of Chronic Traumatic Encephalopathy Research from 1999 to 2019* (62), which shares similarities with our own conclusion. For example, the study found that the USA had the highest number of CTE-related publications, with nine of the top 10 institutions located in America, notably Boston University with the highest number of publications, which is not surprising given its focus on CTE. Cognition impairment is a common and notable manifestation of CTE (27), which could occur in TBI patients of diverse occupations, for example, boxers (49), football players (51, 63), and military servicemen (23). Some CTE patients suffer from cognition dysfunction as the initial manifestation (27). As our study focuses on post-TBI dementia, we cannot overlook the research area of CTE, and studies on changes in cognitive function in CTE should be included in our retrieval results. Our findings demonstrate that research about CTE represents a substantial proportion of the discipline of post-TBI dementia. CTE has emerged as a significant research direction in the field of post-TBI dementia.

### Current controversies and prospects

The consensus amongst the majority of scholars is that TBI is a significant risk factor for dementia. However, generous studies suggest that not all TBIs are likely to be associated with an increased risk of developing dementia. Grasset et al. (8) did not establish such a long-term link between TBI with loss of consciousness (LOC) and dementia or memory decline. Nguyen et al. (9) also did not discover a significant correlation between remote TBI with LOC and the onset of Dementia with Lewy bodies (DLB). And Plassman et al. (10) thought that TBI was associated with non-Alzheimer’s disease dementia but not AD. The relationship between TBI and dementia has gained considerable attention among scholars in this area, and a definite causality between the previous history of TBI and dementia onset has not been established (7). Therefore, future research is warranted to gain an in-depth understanding of post-TBI dementia. Several researchers (4, 38, 64) observed that patients with a history of moderate or severe TBI had a greater risk of developing dementia compared with those with mTBI, which seemed to suggest a dose-dependent relationship between TBI severity and subsequent dementia risk. However, this perspective has been challenged. Stopa et al. (2) proposed that the risk of dementia after mTBI was greater than that of moderate and severe TBI. This inconsistency of findings might be due to the distinctive pathogenic mechanisms by which TBIs of diverse severity participate in the progression of dementia. MTBI without LOC might induce an increased risk of dementia primarily via accelerating brain atrophy, whereas moderate to severe TBI affects more baldly the deposit of Aβ and tau (38). Repetitive TBIs could exacerbate the damage and lead to worse outcomes (1, 65), supporting the aforementioned dose-dependent relationship. However, as with the findings on the implications of the severity of TBI, controversy exists surrounding the frequency of TBI. Perry et al. (5) proposed that repeated TBIs did not result in a significantly increased risk of dementia compared to a single TBI. Godbolt et al. (66) pointed out that neither single nor repeated mTBIs induced a significantly raised risk of dementia. In sum, the nature of TBI could evidently affect its prognosis. It is necessary to carry out additional studies that control for variables and investigate the relationship between TBIs with varying characteristics and dementia. Moreover, it is significant to validate this dose-dependent relationship for a comprehensive understanding of the correlation between TBI and dementia, which would guide clinical decision-making.

CTE is a distinctive neurodegenerative disease characterized by a spectrum of distinguishing pathological features like the unique configuration or distribution of tauopathy, which are different from those that existed in aging, AD, or any other tauopathy (23, 67). Age-related tau astrogliopathy (ARTAG), a form of astrocytic phosphorylated tau pathology, is an age-related alteration that is common in CTE. A few researchers might have mistakenly interpreted ARTAG as a diagnostic pathology of CTE, leading to disputed findings (67). CTE has gradually received much attention for its strong association with TBI, especially rTBIs (23, 54, 67). Nevertheless, a conclusive causal relationship between rTBIs and CTE has not been established yet. The latest article by McKee et al. (67) on neuropathological diagnostic criteria for CTE did support such a causality despite the lack of definitive evidence. Currently, the diagnosis of CTE relies on its specific neuropathological alterations (24, 68), which makes it challenging to diagnose patients with CTE definitely during their lifetime. There is a need for diagnostic criteria for CTE that are more applicable to clinical work. In addition, demonstrating such causality could be difficult due to possible selection bias, limitations of research types (e.g., cross-sectional studies and case reports), and other factors from previous studies. Furthermore, accurately quantifying the lifetime TBI exposure, which is significant and necessary to clarify the unknowns in the field of post-TBI dementia, could also be a huge issue (7, 69). Prospective and longitudinal studies of individuals with a hazard of developing CTE are necessary to overcome these challenges. This can be achieved by incorporating clinical injury exposure metrics, collected through wearable measurement devices, as well as evolving fluid and neuroimaging biomarkers (67, 69).

As previously mentioned, TBI could increase the risk of developing AD, VD, PD, and MCI (4, 5). According to McKee et al. (23), out of 65 CTE subjects with a history of repetitive mTBIs (rmTBIs), 30 (46.2%) exhibited pathological comorbidities, including AD, motor neuron disease (MND), PD, Lewy body disease (LBD), FTLD, Pick’s disease, and progressive supranuclear palsy (PSP). There are many questions we cannot help but ask: why does the same reason (TBI) lead to different outcomes (different types of diseases)? Does it suggest a shared pathogenic mechanism in the onset of these diseases post-TBI? Moreover, TBI appears to cause the most measurable strain and mechanical deformation at the depth of the cortical sulcus and around small vessels (67), where the pathognomonic lesion of CTE (hyperphosphorylated tau) also appears (24, 68). The alterations in the early stages of TBI seem to be induced primarily by initial axon injury, which could arouse and prompt a series of secondary damages, such as neuroinflammation and proteinopathies, leading to an increased risk of neurodegeneration (53, 70). The deposit of TDP-43 (TAR DNA-binding protein 43) and tau in the neocortex, medial temporal lobes, and deeper brain structures might be linked to cognitive, memory, and behavior changes, as well as parkinsonism. And cortical regions with a greater TDP-43 pathological burden might be associated with cognitive impairment in CTE patients (22). It is reasonable to speculate that TBI causes axonal injury across multiple regions of the brain, where the initial axonal injury would contribute to the accumulation of toxic proteins and other secondary injurious responses, further leading to the onset and progression of neurodegenerative diseases, such as dementia. And the different manifestations in TBI patients might reflect impairments in diverse spatial locations of the CNS resulting from similar pathogenic mechanisms (71). However, further research is required to deepen our understanding of post-TBI dementia.

Graham et al. (72) conducted a comparative study on the patterns of neurodegeneration in moderate to severe TBI patients with those suffering from AD. The study spanned a median duration of 2.1 years after the injury. With respect to the brain regions with progressive atrophy, their findings indicated a visible overlap between TBI and AD patients in part of the white matter (WM), predominantly subcortical regions rather than deep WM structures. In contrast, the gray matter (GM) exhibited no significant overlap. And “TBI-specific” areas with significant atrophy included “the corona radiata, the corpus callosum, the corticospinal tracts, and so on,” while “AD-specific” regions included “temporal GM, parietal and occipital cortices,” as well as parts of the WM. The findings of this study suggest that distinct patterns of atrophy occur in AD and following TBI, and TBI could significantly affect the subcortical and deep WM structures. However, the situation is discordant in mTBI. Zhou et al. (73) explored the brain volume change of mTBI patients with an average follow-up time of 1 year and 1 month. They found significantly reduced brain volumes in the anterior cingulate WM, the left cingulate gyrus isthmus WM, and the right precuneus GM in mTBI patients compared with healthy control subjects. And the volume loss in the rostral anterior cingulate WM might be relevant to poor neurocognitive performance. Shida et al. (74) analyzed the effect of mTBI on brain aging, especially the loss of GM volume, based on MRI images around 6 months post-TBI. They observed that the average biological age of brain areas in mTBI patients was 9.2 years older than that of healthy controls. The regional GM most significantly affected by mTBI involved “the short gyri, long gyrus, and central sulcus of the insula.” Rostowsky et al. (75) compared the neurodegeneration 6 months post mTBI with that of AD and observed a significant overlap of abnormal brain regions, in both cases, not only in parts of WM but also in parts of GM, which indicated that the effects of mTBI on the brain might be different from that observed in cases of moderate and severe TBI, further supporting the idea that TBIs of different severity have distinct pathogenic mechanisms. The pattern of neurodegeneration following TBI should be further investigated to fully compare TBI with AD and other neurodegenerative disorders. Additionally, examining TBI patients with favorable outcomes might conduce to identifying protective factors and understanding the progression of post-TBI dementia and other neurodegenerative diseases (69).

## Conclusion

In conclusion, post-TBI dementia has raised great interest among neuroscientists, pathologists, and neurologists, among others, worldwide, and abundant studies, focusing on the field, are being conducted each year. The United States is the dominant country in this area in nearly all respects, including the number of publications, prestigious scientists, and notable institutes. The United Kingdom and Canada also have great influence and involvement in this field. Despite having the second-largest number of articles, the prestige of China underperforms. China should focus on participating in more international research cooperation. The hot spots in this field include “The correlation between TBI and dementia-related alteration” and “CTE.” Moreover, researchers are also focusing their attention on clinical manifestation, therapy, pathology, and pathogenic mechanisms. In summary, the study provides valuable insights into the current status and progression of the discipline, which is expected to inform and improve future research in this area.

## Data availability statement

The original contributions presented in the study are included in the article/Supplementary material, further inquiries can be directed to the corresponding authors.

## Author contributions

HR and L-JH determined the theme of the study. X-ZS, C-QW, and WC designed the study, scrutinized the data, wrote the manuscript, and made the figures and tables. X-ZS and C-QW collected data. The bibliometric analysis-related software and the online platform were operated by X-ZS and WC. Data analysis and interpretation were done by X-ZS, C-QW, WC, HR, and L-JH. WC, C-QW, HR, and L-JH revised the article. Bibliometrics-related guidance was from HR. Medicine-related guidance was from L-JH and HR. All authors contributed to the article and approved the submitted version.

## Funding

The work is supported by the National Natural Science Foundation of China (Grant number: 82201519), and Shanghai Changzheng Hospital under Pyramid Talent Project (YQ684).

## Conflict of interest

The authors declare that the research was conducted in the absence of any commercial or financial relationships that could be construed as a potential conflict of interest.

## Publisher’s note

All claims expressed in this article are solely those of the authors and do not necessarily represent those of their affiliated organizations, or those of the publisher, the editors and the reviewers. Any product that may be evaluated in this article, or claim that may be made by its manufacturer, is not guaranteed or endorsed by the publisher.

## Footnotes

^1^https://www.cdc.gov/traumaticbraininjury/health-disparities-tbi.html

^2^https://bibliometric.com
