# Supplementary material for: An exhaustive analysis of post-traumatic brain injury dementia using bibliometric methodologies
Source: Front Neurol. 2023 Jun 29;14:1165059. doi: 10.3389/fneur.2023.1165059 (PMC10345842; doi:10.3389/fneur.2023.1165059)
Supplement: Supplementary file 1 [file Data_Sheet_1.docx]

| 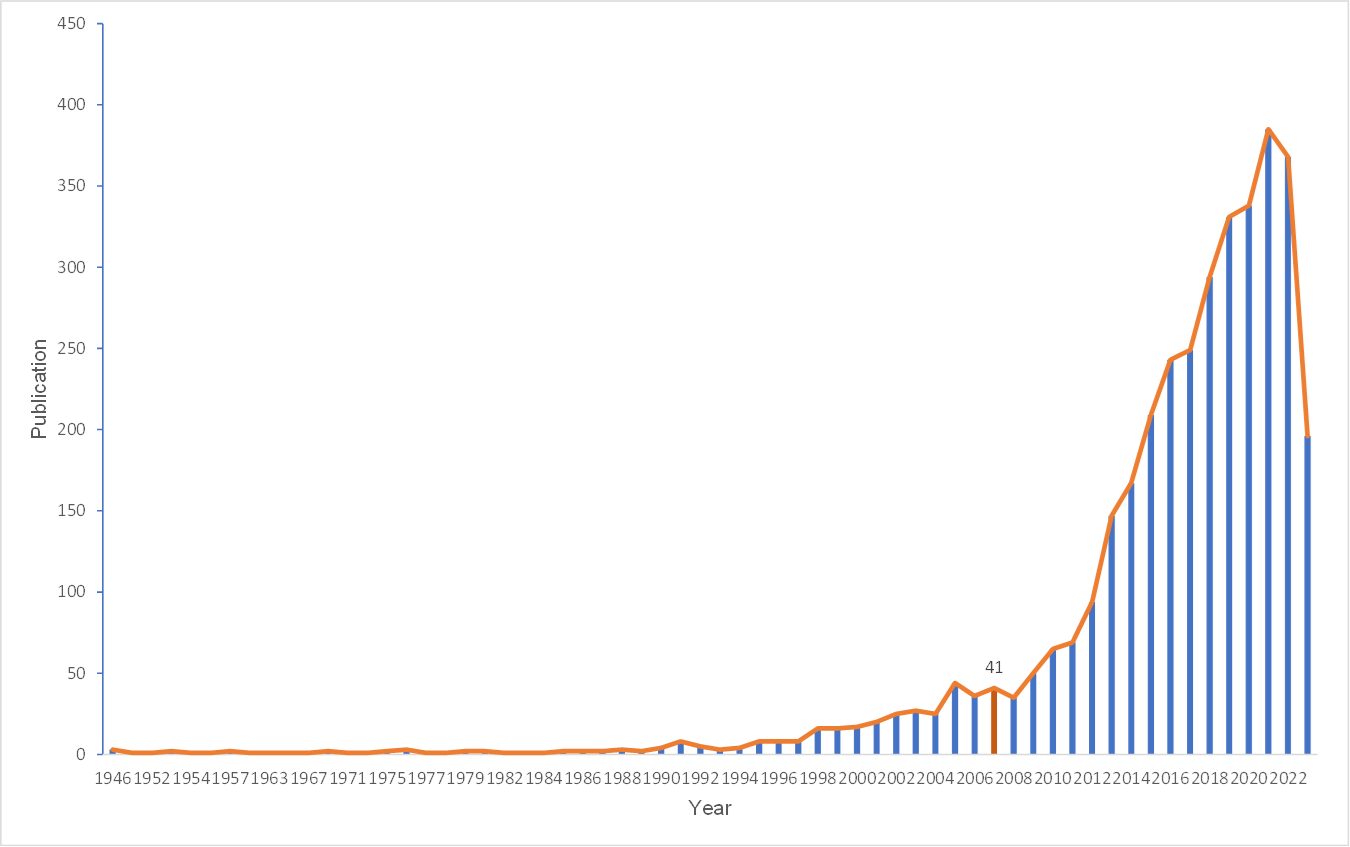  **Supplementary Figure S1**  The number of annual publications in the field of post-TBI dementia. The data was gained from PubMed. |
| --- |

| **A B**  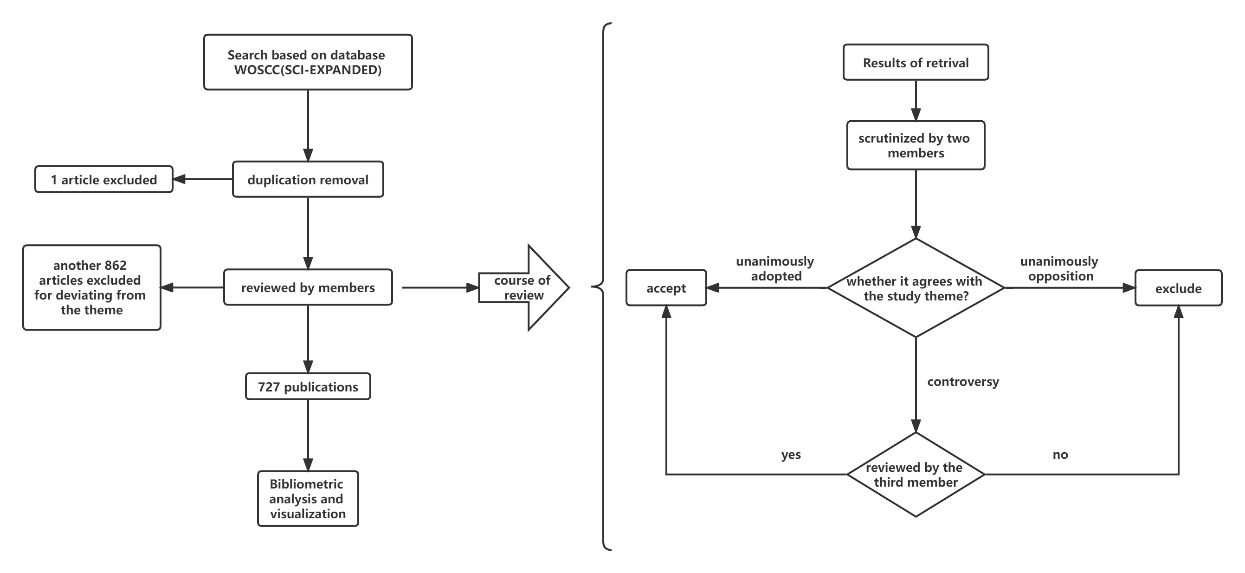**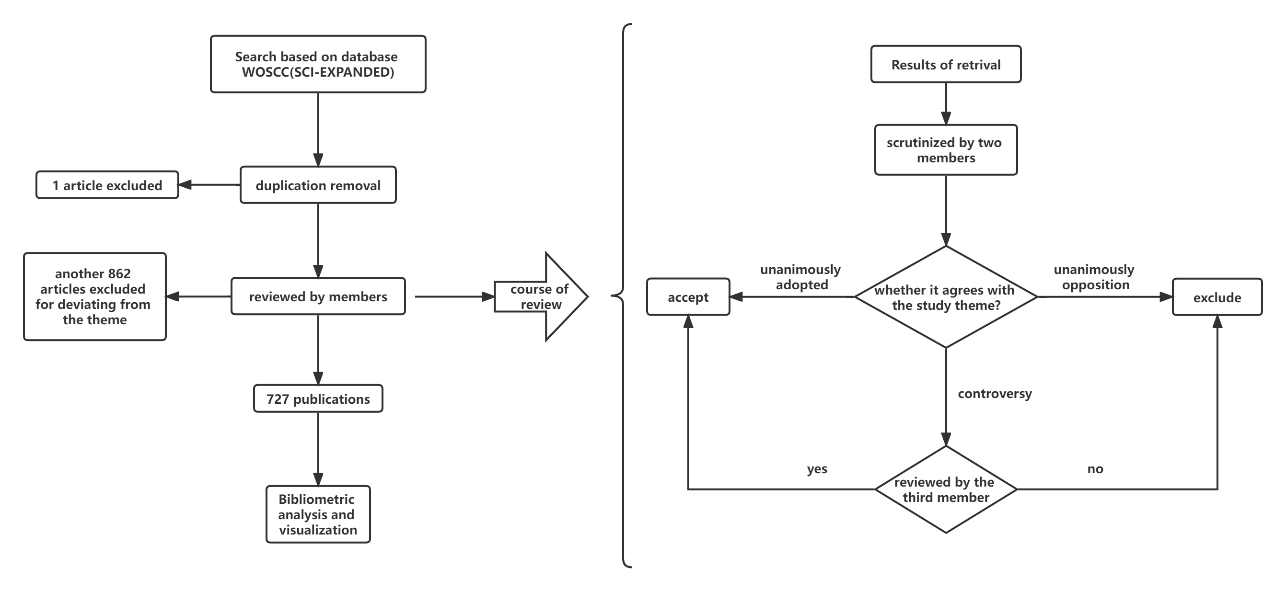**  **Supplementary Figure** S**2**  (A) Flowchart of Data Acquisition. (B) The course of the review. |
| --- |

| \| **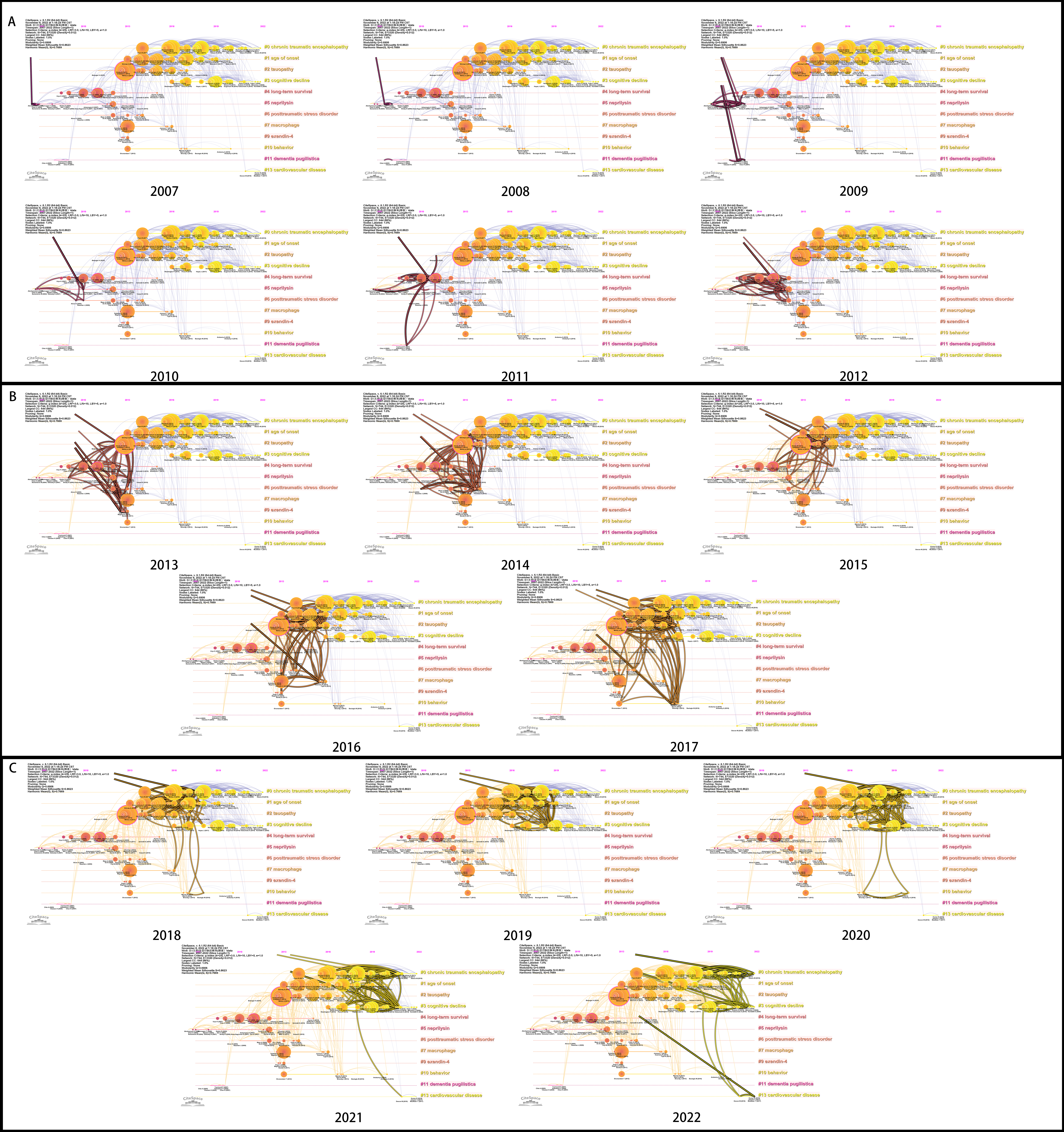** \| \| --- \|   **Supplementary Figure S3**  The history of citations from 2007 to 2022. (A) Quotations between 2007 and 2012. (B) Quotations between 2013 and 2017. (C) Quotations between 2018 and 2022. The figure is obtained from CiteSpace. |
| --- | --- |
